# Medical Diagnosis

**Published:** 1884-07

**Authors:** 


MEDICAL DIAGNOSIS WITH SPECIAL REFERENCE TO PRACTICAL MEDICINE. —A Guide to the Knowledge and Discrimination of Diseases. By J. M. Da Costa, M.D., LL.D., Professor of Practice of Medicine and of Clinical Medicine at the Jefferson Medical College, etc. Illustrated with Engravings on Wood. Sixth Edition, revised. Philadelphia, J. B. Lippincott & Co., 1884.

The fact that this is the sixth edition of this popular work, and that it has been published in several foreign countries is sufficient evidence of its general merit. The work has been revised and much new matter added, making it unquestionably the best work on diagnosis published. No physician's library can be complete without it.

